# Caregivers’ Grief in Acquired Non-death Interpersonal Loss (NoDIL): A Process Based Model With Implications for Theory, Research, and Intervention

**DOI:** 10.3389/fpsyg.2021.676536

**Published:** 2021-04-30

**Authors:** Einat Yehene, Alexander Manevich, Simon Shimshon Rubin

**Affiliations:** ^1^School of Behavioral Sciences, The Academic College of Tel Aviv-Yaffo, Tel Aviv, Israel; ^2^The School of Psychological Sciences and the International Laboratory for the Study of Loss, Bereavement and Human Resilience, University of Haifa, Haifa, Israel; ^3^Department of Psychology, Max Stern Academic College of Emek Yezreel, Jezreel Valley, Israel

**Keywords:** caregiving, grief, attachment, continuing bonds matrix, interpersonal loss, ambiguous loss, bereavement

## Abstract

The number of family members caring and caregiving for a loved one undergoing physical and mental changes continues to increase dramatically. For many, this ongoing experience not only involves the *“burden of caregiving” but* also the *“burden of grief”* as their loved-one’s newfound medical condition can result in the loss of the person they previously knew. Dramatic cognitive, behavioral, and personality changes, often leave caregivers bereft of the significant relationship they shared with the affected person prior to the illness or injury. This results in what we term conditions of acquired “non-death interpersonal loss” (NoDIL). Current approaches to these losses use an amalgam of models drawn from both death and non-death loss. Despite their utility, these frameworks have not adequately addressed the unique processes occurring in the interpersonal sphere where the grieving caregiver needs to reach some *modus vivendi* regarding the triad of “who the person was,” “who they are now,” and “who they will yet become.” In this paper we propose a process-based model which addresses cognitive-emotional-behavioral challenges caregivers meet in the face of their new reality. These require a revision of the interpersonal schemas and the relationships that takes into account the ongoing interactions with the affected family member. The model and its utility to identify adaptive and maladaptive responses to NoDIL is elaborated upon with clinical material obtained from caregivers of people diagnosed with major neuro-cognitive disorder and pediatric traumatic brain injury. The article concludes with implications for theory, research and clinical intervention.

## Introduction

“Bereave,” according to the Merriam-Webster dictionary, is defined as “to deprive of something,” or “to take away a valued or necessary possession, especially by force” (Merriam-Webster, 2020). Although this definition is rather broad and inclusive in nature, bereavement, mourning and grief are still recognized and understood as responses to the death of a loved one, also referred to as an attachment figure. Such term derives from Bowlby’s attachment theory, which stresses the importance of these relationships for physical and psychological survival and well-being (Bowlby, 1969, 1973; Cassidy and Shaver, 2016; Lahousen et al., 2019). Accordingly, grief and mourning are framed as responses to broken attachment bonds (Bowlby, 1980; Jordan, 2020).

In recent decades, there is a growing recognition that other life-altering events that do not involve death, can also leave family-members bereft of an important relationship, due to dramatic changes to their affected loved one. Those can arise from a number of medical and psychological conditions, typically ongoing in nature and do not allow for closure, hence resulting in what we term as, conditions of *acquired “non-death interpersonal loss”* (NoDIL). Namely, we are referring to that class of loss that describes a change in the relationship driven by the condition where the attachment figure is no longer who he or she had been prior to dramatic change. Instead, the person to whom the griever is connected has dramatically changed in essence -in fact, the person is literally “alive” but effectively “gone.” Unlike divorce, for instance, where the other person is basically the “same person” but the relationship has effectively ended, in an acquired NoDIL, the relationship may or may not continue, but it is with an “altered” person, different than the one known previously.

This type of loss is an increasingly common occurrence, constituting a *“silent pandemic”* which greatly impacts modern society. Advances and developments in medicine have led to increased numbers of people surviving what had once been fatal injuries, illnesses, and overall increased life expectancy of elder population (Olshansky, 2018). Such trends resulted in a larger portion of society experiencing pronounced brain impairments due to traumatic brain injury (TBI), stroke, Parkinson’s, Alzheimer’s disease among other various medical conditions and chronic illnesses. Under these circumstances, many individuals also undergo significant cognitive dysfunction involving intellect, communication and behavioral changes alongside personality alteration (Collings, 2008; Jordan and Linden, 2013; Bodley-Scott and Riley, 2015; Riley, 2016; Galimberti and Scarpini, 2018; Li et al., 2020). These extract a toll from the afflicted and their families as well. The number of family members caring and caregiving for their child/parent/spouse experiencing these conditions is likewise on the rise. In the United States alone, this number has increased by 9.5 million from 2015 to 2020 and is now 53 million (The National Alliance for Caregiving Mission, 2020). Changes in patient-care practices along with the emotional and financial expense of placing a family member in long-term care facilities, oftentimes lead many caregivers to prefer home-care placement as an arrangement to cope with their loved ones’ life-situation. Either way, in addition to the stress and burden which caretaking may require, many caregivers also experience and grieve the “interpersonal loss” encountered. The person they once knew has become someone whose personality and behavior may be barely recognizable even as the physical body remains relatively intact. The latter is already known to adversely impact caregivers mental and physical health (Volicer, 2005; Pagani et al., 2014; Tzuang and Gallagher-Thompson, 2014; Saban et al., 2016; Gérain and Zech, 2019; Watson et al., 2019).

Similar to the loss of highly significant relationships following death, which set into motion the grief and mourning processes, recent studies have showed the presence of grief among caregivers in various conditions. These include caregivers of people suffering from major neurocognitive disorder (Dementia) (Chan et al., 2013; Lindauer and Harvath, 2014; Liew et al., 2020; Meichsner et al., 2020; Manevich et al., 2021), Brain Injury (Marwit and Kaye, 2006; Petersen and Sanders, 2015; Yehene et al., 2021), and Disorders of Consciousness (de la Morena and Cruzado, 2013; Yehene et al., 2020), etc. However, despite the significant contribution of these studies, less is known about the mechanisms and processes underlying psychological reactions to such acquired NoDIL and what is the nature of the grief and mourning that follow in their wake.

**FIGURE 1:**
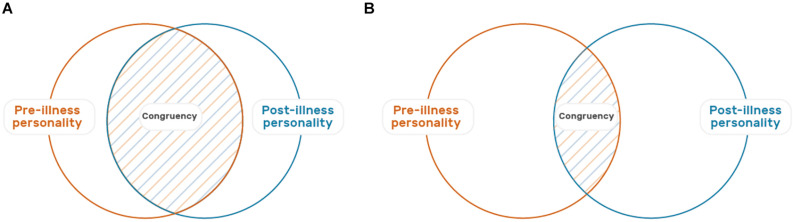
Illustration of the personality changes and relationship challenges associated with Acquired Non-Death Interpersonal Loss (NoDIL). In these two Venn diagrams, the left orange circle represents the personality of the loved one before the illness, and the right blue circle is their current personality. The dotted area in blue and orange is the degree of overlap and continuity between the two. The overlap in diagram **(A)** is greater than that of **(B)**, signifying greater congruence between past and present personality.

Accordingly, the present article aims to provide an in-depth analysis of the grief and mourning that often accompany acquired NoDIL. We do this by clarifying and specifying important aspects of the sources, processes, and various outcomes of dealing with these losses. While comparison with interpersonal loss via death contributes to these analyses, we also consider the important differences that exist between these classes of loss, as well as the implications of these differences. Given the millions of caregiving family members and the professional healthcare providers working with them, attention to these issues is highly relevant. In this regard, the paper is geared at both health care professionals and anyone else who finds this topic of relevance including caregivers. At the same time, when considering the field of NoDIL, our aim is to go beyond the familiar concept of burden (Chou, 2000; Volicer, 2005; Aitken et al., 2009; Covelli et al., 2016; Doser and Norup, 2016) and to expand beyond a consideration of well-being and biopsychosocial adaptation (Bleijlevens et al., 2015; Magnani et al., 2020). Specifically, we consider what caregivers grieve and the extent to which they “rebalance” and maintain the relationship with the affected person (Soeterik et al., 2018; Yehene et al., 2019a,b; Lond and Williamson, 2020). In that respect, understanding how caregivers find their way amongst the memories of “who the person was” and to living now with “who they are now” is highly important (see Figure 1). Such identification and specification of the psychological processes NoDILs entail has the potential to advance theory and research as well as supportive interventions aimed to promote caregivers’ emotional well-being in clinical practice.

## From Detachment to Continuing Bonds in Bereavement Due to Death

Freud began his 1917 article on Mourning and Melancholia by describing mourning as occurring following the death of a person or the loss of something critically important to the griever. Freud’s highly influential article led to decades of theory, research and practice that formulated the processes of grief and mourning as essentially concerned with letting go of the relationship with the deceased or to something highly valued (Freud, 1917). With the benefit of hindsight, the field has moved away from the idea of de-cathexis or the withdrawal of emotional investment in the deceased to one that favors a reworking of the relationship and the continuation of the emotional connection to the memories of the deceased (Rubin, 1992; Klass et al., 1996; Klass and Steffen, 2018).

Along with this paradigm-shift in understanding response to death, recent models of such bereavements allude to the “non-linearity” process of grieving and the connection with the deceased throughout life. Unlike the stages-model presented by Kübler-Ross (1969), in these models the reworking of the “continuing bond” with the deceased is a central grief related task. For instance, The Two-Track Model of Bereavement (TTMB) (Rubin, 1981, 1999) addresses not only the bereaved biopsychosocial functioning and coping with the demands of life post loss (Track I), but also the nature of the bond with the deceased and the integration of the “death story” (Track II). In the complex and multi-tiered process of responding to loss, one or the other aspect of functioning and relationship to the deceased can be at the forefront of consciousness, but understanding how loss is being processed requires monitoring both tracks of the bereaved’s experience (Rubin et al., 2012, 2020). Another model is the Dual Process Model (DPM) of coping with bereavement which emphasizes how grief experience unfold via an oscillation between focus on the relationship with the deceased (loss-orientation) and alternate focus upon tasks of everyday life and distractions (restoration-orientation) (Stroebe and Schut, 1999, 2010, 2016). Such reworking of the continuing bonds with the deceased is often a focus of the constructivist approach which highlights the importance of meaning making in coping with loss (Neimeyer et al., 2006; Neimeyer, 2016; Smid, 2020).

Shared by these models and by the majority of current approaches to adjustment to loss and bereavement is the understanding that the physical absence of the loved one in death elicits a process of grief and adjustment (Malkinson et al., 2000; Bonanno et al., 2011; Galatzer-Levy et al., 2018). Traversing these processes result in adaptation to their changed life circumstances with the understanding that the continued bond with the deceased fulfills critical adaptive elements for adjustment post loss (Rando, 1984; Klass et al., 1996; Malkinson, 2007; Stroebe et al., 2008; Worden, 2018). Current diagnostic approaches to complications in the bereavement process have particularly targeted the prolonged grief reaction (World Health Organization [WHO], 2018; Boelen et al., 2020; Killikelly and Maercker, 2020; Rubin et al., 2020) with criteria focused on both the yearning for the deceased together with maladaptive behaviors and impairment in the tasks of life. However, such complications in the bereavement processes may be inherently embedded in certain medical conditions involving NoDIL, resulting in prolonged grief reactions (Boerner and Schulz, 2009; Zaksh et al., 2019). Diagnostic criteria do not include or contrast this type of loss with bereavement despite the many elements shared in these two conditions. We will address this point later.

## From Stress and Burden to Grief and Mourning in Non-Death Bereavements

To date, current models of bereavement and loss have not sufficiently provided applicable and clinically relevant conceptualizations concerning the process of managing the relationship with the altered person, where the person has dramatically changed but the relationship continues. In contrast to bereavements due to death, in acquired NoDIL, the physical presence of the “altered” person coexists with the awareness that the essence of the person who had been there before is now “gone.” In those many cases where the “lost” person is never to return, the acceptance of the “new” person will continue alongside the memory of the “old” person. The contrast between “old” and “new” has the potential to create significant complications in the grieving process and to make the loss gather significance over time. This is due to the conflicting and often mutually exclusive attachment schema (i.e., schema) regarding who the person is and how to relate to him or her. Thus, to promote psychological adaptation, for the **grieving caregivers**, some manner of processing and re-working of the relationship with their loved one, while he or she is still alive, is a daunting but necessary task.

For many decades, caregivers’ psychological reaction to non-death losses was primarily understood through the lens of how their loved one’s condition affected their overall well-being (Lim and Zebrack, 2004). Specifically, research has largely focused on the stress and burden characterizing caregivers’ daily-life (Lazarus and Folkman, 1984; Pearlin et al., 1990; Zarit and Femia, 2008; Giovannetti et al., 2013; Tan et al., 2019) along with the personal losses resulting from their newfound responsibilities (loss of autonomy, relinquishing of anticipated future plans, etc.) (Pertl et al., 2019). Changes to their belief system regarding the world and the self were also noted (“assumptive world”) (Janoff-Bulman, 1992; Parkes, 2001; Harris, 2011). Conversely, despite accumulated evidence regarding grief reaction among caregivers (Petersen and Sanders, 2015; Lond and Williamson, 2020), writings and conceptualizations concerning their loss experience, in particular those related to the interpersonal sphere (Rubin et al., 2019), remained limited in scope over the years.

Almost 20 years ago, Bruce and Schultz (2001) coined the term *“non-finite loss”* to describe an enduring loss, precipitated by a negative life event after which the source of the loss continues to be present. This term was initially used in cases of children with developmental disabilities or chronic diseases (Hobdell, 2004; O’Brien, 2007; Whittingham et al., 2013; Bravo-Benitez et al., 2019). Such loss manifests itself gradually, and is often characterized by a sense of ongoing uncertainty, repeated adjustment and accommodations, with an unforeseen end (Harris, 2011, 2019). Emotional reaction to non-finite loss has often been termed *“chronic sorrow”* (Olshansky, 1962; Coughlin and Sethares, 2017). This term refers to a set of pervasive, profound, enduring grief reactions, that are constantly triggered by painful discrepancies between present reality and what was hoped and imagined for the future. A similar notion was rendered as *“living-loss”* and also stressed the ongoing nature of the loss experience (Roos, 2002). To date, these influential concepts are still used primarily to describe parental experience in cases of child disability or chronic illnesses, and their application to cases of acquired rather than developmental loss, is still scarce. In addition to their limited use, these concepts addressing non-finite loss and chronic sorrow do not consider the relational sphere with the attendant need to consider how the attachment bond with the child is affected by the discrepancies between the “hoped for” and the “lived” developing child which is driving the chronicity of the sorrow.

Another central and influential contribution to the field of non-death loss is the work of Pauline Boss on *“Ambiguous Loss”* (Boss, 1999, 2007, 2010). Boss’s framework addresses the relational sphere as when she considers discrepancies between physical and psychological absence. In the model “goodbye without leaving”/”leaving without goodbye” (Boss and Yeats, 2014), the authors refer to cases of physical presence but psychological absence (i.e., coma, dementia, mental illness) or physical absence but psychological presence (i.e., kidnapping, missing in action). According to Boss, such cases of incongruity involve great ambiguity regarding whether the person is still part of the relationship in light of the hope that they will reappear as they were. Being a condition without the finality of death, caregivers’ grief remains frozen, non-legitimized and “disenfranchised” by society (Doka, 2002, 2008; Boss and Carnes, 2012). However, while Boss’s “goodbye without leaving” concept is relevant for the discussion at hand, it does not sufficiently address the mechanisms or the processes in which the griever is challenged.

In our view, in cases of NoDIL, much of the loss experience is rooted in the discrepancy between “who the person was previously” and “who they are today” and “who they will be in the future.” This mix of psychological representations requires a process of “working through” to come to terms with their predicament. Caregivers need to pave a way through an “interpersonal limbo” in which they are not only required to say “goodbye” to what has been the nature of the person and the relationship in the past. They are also required to say “hello” to a dramatically changed person and to manage a relationship that cannot continue unmodified.

While Boss’s work largely contributed to the focus on non-death loss, her framework leaves unspecified the complexity of various conditions in which old and new aspects of the person are active in the mind of the family member. Boss refers to who was “lost” and does not sufficiently focus on “what was lost” with regards to that person. Accordingly, she largely emphasizes the management of ambiguous loss within the family alongside strategies for coping (Boss and Yeats, 2014). The mechanisms by which family members balance and re-construct these competing schemas of their altered-other have yet to be outlined. Therefore, clarifying these processes can largely contribute to the progressing field of non-death loss.

At this point, we advance a conceptual framework outlining the mechanism underlying this type of loss and the cognitive, emotional and behavioral aspects it brings about. Such a framework should also take into consideration the impact of the varying nature of the illness and prognosis, helping clinicians to identify nodal points that can elicit maladaptive responses and undue suffering.

## Death vs. NoDIL—Introducing the Notion of Open vs. Closed System

As mentioned earlier, the heart of the bereavement-due-to-death experience, however, is not the requirement of the griever to manage the demands of life for this crisis as in any other. Rather, it is the reworking of the continuing bonds with the loved one (Rubin et al., 2012, 2020). What had been the connection to a living person with possibilities and experiences of interaction must accommodate a new reality where the connection in the physical sphere has ended but continues to exist in the psychological realm only via memory and imagination. The demands of the reworking of the relationship to the deceased, therefore, become part of a predominantly *closed system*, where no interaction exists in the physical sphere and hence, there is no incoming interpersonal input to be processed. In this respect, one reorients from the external world of no connection to the inner world of memory and affect of an internalized relationship.

Conversely, in non-death losses, the ongoing relationship to the loved one is in its stead, an *open system* where the still living albeit ‘‘altered’’ person continues to be part of the caregiver’s reality. Thus, the interaction *continues* in the physical sphere, and that continuation means that the **continuing bond** is not bound by the relationship in the past only, as in the aftermath of death. Rather, it operates as an *open system* in which *new interpersonal input* approaches reality and is being continuously integrated. Thus, the ongoing connection to the altered-loved one is influenced by the nature of the additional interactions that began with the life-altering event and continues onward, often with great uncertainty and ambiguity. Naturally, *new interpersonal input* can vary in quantity and quality per medical condition and its prognosis. Also, it may or may not contradict existing knowledge and schemas about the affected person’s personality, the nature of past relationships and the envisioned future prior to the event^1^.

**FIGURE 2:**
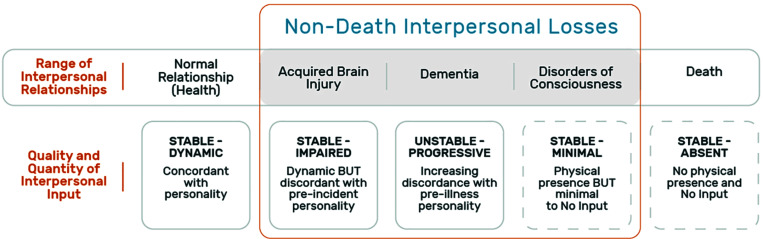
The varying quantity and quality of incoming interpersonal input in the physical sphere in a range of interpersonal relationships. This figure presents a range of interpersonal relationships and the associated incoming information that characterizes each. The top row presents the types of relationships: normal relationship (health), non-death interpersonal losses (acquired brain injury, dementia, and disorders of consciousness as common examples), and finally death. The bottom row describes the quality and quantity of the incoming interpersonal input that characterizes each relationship.

Figure 2 illustrates and summarizes this idea on a wide spectrum of prototype conditions from normative healthy relationship, through medical conditions to death.

To summarize, the interaction with the loved one prior to the change has ended and a modified relationship is developing. Therefore, understanding the way that these processes are experienced within the griever, may shed light on the way bereavement and non-death losses unfold over time.

## NoDIL—The Continuing Bonds Matrix Model

**FIGURE 3:**
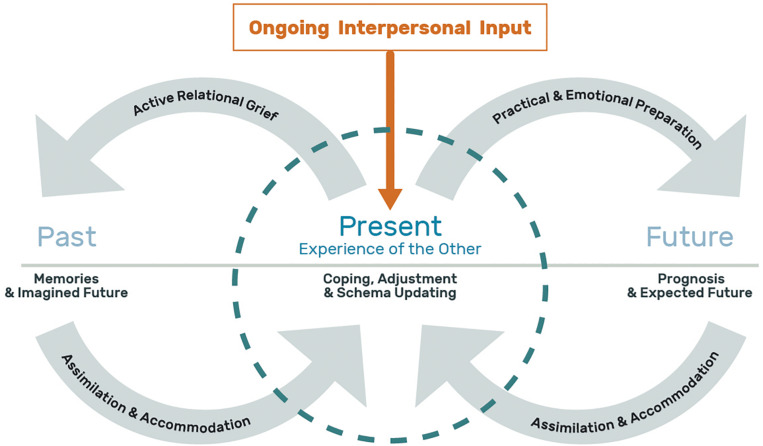
A process-based model of continuing bonds matrix reconstruction and coping in Acquired Non-Death Interpersonal Loss (NoDIL). This figure depicts the NoDIL model where the center circle represents the present experience of the loved one and is placed midway between the time dimensions of past and future. The “past” on the left includes the memories of the person as they were prior to the illness/injury event along with what had been the imagined future of that time. The “present” encompasses the contemporary perception of the significant other along with the tasks of coping, adjustment and schema updating. On the right, the “future” represents the information and experience driven by prognosis and the expected future. Linking the three time dimensions are gray arrows (forming an infinity like symbol) that represent the dynamic interactive processes between them: Active relational grief for the “past” and its assimilation and accommodation in the “present” schema, along with emotional and practical preparation for the “future” and its assimilation and accommodation in the “present.” The ongoing interpersonal input in orange, appears within a rectangular frame with an arrow facing downward indicating a continuous stream of incoming information from the loved one that impact the “present” schema. Lastly, the open spaces in the circle’s perimeter stress the permeable boundaries of the present time frame.

The following section of the article will be devoted to presenting a process-based model of continuing bonds matrix reconstruction and coping in NoDIL. This model constitutes an integration and expansion of current theoretical, clinical and empirical knowledge as reviewed in this paper, and strives to address issues that have previously remained insufficiently answered. The model described below consists of three main components: ongoing interpersonal input and its quality; 3-dimensional schemas of past, present and future; and cognitive, emotional, and behavioral changes required for adaptive coping. Figure 3 and accompanying text put forth this process-based model.

First, and based on the above differentiation between closed vs. open systems, NoDIL is an open system that includes an ongoing flow of incoming information from the altered loved one, which is the focus of this experience and its uniqueness. The informational input of this kind leads to branching out in the cognitive-emotional domain. The memory representations of the person as they had been in the past prior to the altering event(s), exist in contrast to how they are today, or are expected to be in the upcoming future. That is, a continuing bonds matrix with and of the loved one that has undergone a personality and functional change due to an acquired medical condition.

Potential disparities between the schemas of the past, present and future may lead to dissonance and an increase in the degree of distress experienced by the individual, and therefore require them to perform a number of parallel and simultaneous processes to minimize the tension caused by these gaps. These processes can be conceptualized by a mechanism of feedback loops of assimilation and schema accommodation at the cognitive, emotional and behavioral levels, in light of the ongoing new information received from the loved one.

Schema updating is done in reference to three dimensions of time. Namely, accommodation of the present schemas in connection to the relationship as it was in the past, and updating the present schemas in light of the future to come. In other words, the personal and functional change that has taken place in the loved one requires the family member to mourn and grieve the loss of the affected person as they were in the past and were imagined to be before the aforementioned change. Accordingly, caregivers are required to integrate and re-construct past representations with present ones. Additionally, the medical prognosis requires emotional and practical preparation for the future to come from the caring and caregiving family member. That is, coordinating expectations and taking preparatory actions regarding the future relationship in the face of the disease. These processes can also be influenced by the quantity and quality of the incoming information due to the medical condition of the patient, the personal characteristics of the caregiver, as well as the nature of the pre-morbid relationship.

Given the challenges described above, schema boundary permeability is a factor of great importance, so that schemas must be sufficiently flexible, in comparison with rigid boundaries that do not allow for schema updating and may contribute to difficulties in coping. In other words, maladaptive coping is conceptualized as deficiency in adaption and inflexibility of schema modification. Here, the person tries to interpret and force the incoming information to conform the “old” schemas, without being able to modify them sufficiently so as to incorporate this new information into and create a new “hybrid” schema. Moreover, the activation and predominance of the “old” schema, manifest in preoccupation (such as rumination on the relationship that existed before the loss) or avoidance (essentially shutting out information or not integrating it into one or more dimensions of time) serve to disrupt the process of adjustment and thus contribute to the individual’s distress.

## Clinical Illustrations

To illustrate the concepts we have been discussing, we now turn to vignettes of relevant clinical and research material that clarify and illustrate the analyses we have put forward. To minimize variability due to gender (Doka and Matrin, 2010) and familial-role differences in the grief process, we have focused on illustrative adaptive and maladaptive responses for the same familial-role and genders^2^.

Ruth’s son sustained traumatic brain injury 2 years ago at the midst of his adolescence:

*“I miss him a lot [*…*]. To his previous abilities and his stubbornness. I am willing to give everything now so he would return to the way he was and the connection we had. His brothers and sister also remember the way he was in the past and the way he is now and this gap drives you crazy and doesn’t let go. It’s in front of you. He could do everything he wanted and now he is like a shadow of his former self. The total opposite. He speaks loudly, very repetitive, childish and needy although age wise he is approaching adulthood. He follows me everywhere. He is like a Robot [in his gait and movements]. [*…*] What happened to us is a terrible thing that no parent can accept! People think that he stayed alive and survived the accident. They don’t understand my own inner experience despite the fact that he stayed alive. I fall into despair. Will I ever be able to see him settled in his own place and live independently? There is no single day that goes without this feeling coming up and me trying to push it aside.”*

Rachel, married for more than four decades to a husband diagnosed with Alzheimer’s disease for the last 5 years:

*“I am not able to accept and hold onto the fact that the man I knew in the past has faded away in front of my eyes [*…*]. It is so hard to digest that his outer appearance remains unchanged—like just the shell, while the inside is no longer there [*…*]. Little by little his personality changed completely—from a gentleman to a coarse and rude fellow, and I changed from a spouse to a 24/7 caretaker [*…*]. I am constantly remembering the life we had before and our plans for the future before this cursed illness entered our lives. It simply shatters me into fragments [*…*]. Truthfully, it only gets worse every day, and I don’t know if I can manage to stand it very much longer. I simply feel exhausted, worn out, angry and hopeless.”*

If we utilize the prism of the model described above, the common feature that makes the experience so difficult for both mother and spouse clearly emerges. In both of these cases, the dramatic personality alteration of the loved one results in a steady flow of incoming interpersonal input that continuously clashes and contradicts with the schema of the past person that they both once knew. The different quality and quantity of information generated in their ongoing interactions with their altered loved one (serving also as a daily reminder of the loss), constantly re-activate the schema of the past person as he had been. For both Rachel and Ruth, the inevitable comparison of the “old” person and relationship with the current ones becomes a continuing aspect of their psychological experience. Their constant remembrance and longing for what was in the past and how their future “should” have been, before it was shattered by the medical condition, causes great distress and relational active grieving. Such intensity magnifies their conflicted and troubled experience, making it difficult to integrate the loss into their life-narrative. This also affects how they approach the demands of the present and how they relate to the expected-futures with their loved ones. In other words, both Rachel and Ruth struggle unsuccessfully to assimilate the alternations that have taken place in their loved ones and accommodate existing schema to better fit the “new” states of their loved one. This state of affairs can be conceptualized as relatively rigid and with impenetrable boundaries that hamper schema updating, thus impeding the process of coping and adjusting to the reality of loss.

In the language of psychological schema, the schema of the past son and husband are significantly separated from the current schema of who they now are. Inflexibility of schema modification processes do not allow to reconstruct an integrated perception of the current person and relationship and therefore, past perceptions remain “encapsulated” in the shadow of the loss resulting in an acute [and prolonged] grief reaction. Thus, it is not only the challenge associated with *burden of care* that is the source of their difficulty, but it is also the *burden of grief* that is so painful and unbearable.

We now turn to two additional cases where the process is experienced differently.

Joan’s son had sustained traumatic brain injury few years ago, just before entering adolescent:

*“There are many thoughts about [child*’*s name] before the injury and it really hurts. I also think a lot about his future—what will be with him. Today when I sit with him to do homework I remember how he was in the past and how easy it was. I used to have a brilliant child with unique characteristics and values. He was born with a clear developmental pattern and suddenly it all shatters, it’s all gone [*…*]. Nowadays, I need to cope with the acceptance of the situation and with the fact that [child*’*s name] is a different child. The only difference is that at the beginning I didn’t want to believe that it’s forever and for the long run [*…*]. Things may improve further but won’t change in essence. For me as a mother, I always have to go through processes in order to understand who this child is and to ‘learn’ about him anew. Sometimes I also get to discover his sense of persistence and his ‘old’ good heart. My husband still struggles and doesn’t understand this. He thinks he will return to the way he was before. I really want him to become an independent normative adult, but I don*’*t live in illusions.”*

Miriam, married for almost four decades to a husband diagnosed with Alzheimer’s disease 5 years ago:

*“[husband*’*s name] was my anchor and strong support, and now that has reversed. Today most of the time I feel like the mother to a little child rather than a spouse [*…*]. I take very good care of him because I haven’t forgotten our past together. He is the biggest love of my life, and I have no doubt that if the situations were reversed, he would do the same for me. We always had this mutual trust and belief and even today, I know that although he does not always remember who I am, he trusts me very much, and I feel secure in my ability to help him, even though it comes at a great personal cost to me [*…*]. True I lost many things over the last few years, but the basic love remains, and at times, on a particular moment in my day, I remember that and it gives me the strength to go on.”*

Looking at these two vignettes, the differences from the earlier cases are prominent. Perhaps the most significant of these differences is that along with the incoming interpersonal information, there is a softening of the schema-boundaries between the loved ones as they had been and as they are now. The constantly changing reality they both must face, brings to the forefront encounters with persisting behavioral and functioning changes of the loved one. In a sense, each encounter gradually penetrates, modifies and extends the internal representations of the now compromised loved one. This can be conceptualized as schema boundaries that are flexible enough in a way that allows for updating of existing schema. Assimilation of present reality and capabilities also serve as a basis for accommodation processes. This is evident in both caregivers’ recognition that it is them who need to “change” so to better interact with their altered loved one and cope with the situation. Despite ongoing emotional hardships and the time it might take to arrive at such realization, this process eventually enables both the “old” and “new” representations [of the relationship and the person] to be integrated and co-exist within one whole single modified-schema. In this way, the myriad of multiple memories, emotion and attachment are not categorized as belonging to two distinct and discontinuous separate individuals as was demonstrated in the first two cases.

In the material presented heretofore, there are several ways of suggesting this integrated continuity. For Joan, it is reflected in her ability to hold on to past qualities she still identifies in her child along with attending to newfound positive qualities post-injury, as well as in her willingness to learn how to deal with the changes set in motion within him following the accident. In the case of Miriam, it is a product of holding the core of mutual commitment to each other that transcends the personality changes that have overtaken her husband. Collectively, for both Joan and Miriam, the recollection of benevolent characters and the relationship as it once was, also constitutes a resource of emotional refueling for their ability to provide their loved ones with care in the present, despite their own personal losses and costs. Lastly, it also seems that the “illness/accident story” is becoming better integrated into their life-narrative as descriptions are accompanied by movement that carries a *developmental quality*. Together, despite continuous sense of agony, these processes help them approach the future with less acute and intensified emotions and with more realistic expectations accompanied by practical adaptations.

## Overview of the Vignettes

The four Vignettes presented hereby alongside their analysis underscore a number of points:

• **Open System and Schema Modification:** The incoming new interpersonal information in these “open systems” of ongoing relationships with dramatically changed loved ones, serves as a major determinant of who the current person is perceived to be. Ultimately, however, it is about how the continuing bonds and perceptions are being integrated, namely, how the entire schema of the currently perceived person is modified, that is most important.
• **Subjectivity of the Loss:** Although the degree of change may vary and at times be greater than the sum of the continuation, it is the magnitude of subjectively perceived change or incompatibility of the “old and new” versions of the attachment figure that greatly determine how caregivers will eventually respond and cope. In other words, the subjective elements of the experience may often outweigh the objective characteristics of the affected family member and the magnitude of change involved.
• **Schema Reconstruction and Psychological Outcome:** Within the continuing bonds matrix, schema updating allows for the attachment to the person their loved ones had been, to soften the distress at who they had become. Conversely, in maladaptive process, past representations of the loved one may serve as painful reminders of what has been lost and increases the distress experience within the continuing bond matrix.
• **Islands of Safe-Haven**: Integrated continuing bonds serve as a positive source of connection that enhances family members’ ability to better relate to and care for the loved one. In those cases, where the link between the person “who had been” and “the person who is now” is sufficiently flexible as well as able to retain the experiences and memories of the positives in the relationship, a balanced experience can be managed.

## Concluding Remarks

The present paper addresses a lacuna on the field at the intersection of death and non-death losses. To date, cases of non-death losses are understood mainly via grief models addressing losses due to death, together with unique conceptualizations in the field of non-death loss emphasizing their ongoing chronic nature. Despite their utility, these conceptual frameworks have not adequately addressed the processes occurring in the interpersonal sphere between the grieving family member and the significantly changed person who’s behavioral and psychological characteristics are dramatically altered from what they had been prior to the accident or illness. In this paper we have proposed a framework that describes and elaborates on the psychological challenges experienced by those who are in close relationships with persons so radically changed. Irrespective of the caregiving burden that may be involved, the psychological bonds and cognitive-emotional representations of the affected individual and the relationship with them require revision and transformation involving grief and adaptation to the new reality.

In our paper, we have stressed the following:

• Interpersonal loss has many manifestations. Our focus on acquired NoDIL addresses those cases where the loved one has undergone major changes that greatly impact the relationship between the affected individual and his or her loved ones. These changes stem from many sources including stroke, physical trauma, dementia, mental illness and any source of dramatic, non-temporary and wide-ranging change in personality and function.
• NoDIL differs significantly from the challenges, grief and mourning associated with the death of close family members. The field of thanatology now stresses the maintenance of the emotional connection to the deceased as a significant aspect of adaptive response to loss by death. The concept of “continuing bonds,” however, needs further specification to understand its applicability to the field of non-death losses as we have outlined here.
• In cases of significant deterioration due to NoDIL much of the loss experience is rooted in the discrepancy between “who they previously were” and “who they are today,” and “who they are to be in the expected future.” Caregivers are faced with the grief over the loss of the person and the relationship known in the past, even as they are challenged to determine what is possible and acceptable to themselves in the present relationship.
• In death, the connection in the physical sphere has ended but continues to exist in the psychological realm only via memory and imagination, thus the relationship becomes part of a predominantly closed system, where no incoming interpersonal input continues to arrive. Conversely, in NoDIL, the ongoing relationship to the “altered” loved one continues within an open system in which new interpersonal input is being continuously encountered. This leads to a situation of contrast between the competing experiences of the affected family member as they were in the past and as they are in the present.
• A process-based model of continuing bonds matrix reconstruction and coping in NoDIL was proposed. This model consists of three main components: ongoing interpersonal input and its quality; schemas of past, present and future; and cognitive, emotional and behavioral tasks that a person must perform for adaptive coping.
• Ultimately, the ongoing revision of the attachment bond redefines the representations of the lost loved one, the identity of the caregiver and their joint life story.

The theoretical, clinical, and research implications of these overlapping and simultaneously dichotomous experiences of the relationship with the loved one, have great significance for understanding how acquired NoDIL unfold over time. Future empirical investigations with qualitative and quantitative research methodologies as well as clinical studies are needed to further determine the utility of the model and its contributions. Ultimately, clinical practice and ongoing research focusing on the way in which close family members deal with reconciling the relationship to the person who is with person who had been can expand our understanding and our clinical work with families dealing with this increasingly common life experience.

## Data Availability Statement

The original contributions presented in the study are included in the article/supplementary material, further inquiries can be directed to the corresponding author/s.

## Author Contributions

All authors listed have made a substantial, direct and intellectual contribution to the work, and approved it for publication.

## Conflict of Interest

The authors declare that the research was conducted in the absence of any commercial or financial relationships that could be construed as a potential conflict of interest.

## Footnotes

1 This distinction (open vs. closed) is a heuristic one since there are many avenues by which new information is actually received in death-loss (e.g., access to social media of the deceased, input from individuals that can occur at any point in time). Nevertheless, in NoDIL this incoming information is continuous and received from the altered-person themselves.

2 Vignettes presented in this paper were adapted from two research projects on caregivers’ grief in pediatric acquired brain injury and Dementia. For both protocols institution review board (IRB) approval and consent were obtained. Caregiver’s verbatim were de-identified by using pseudonymization, and by masking patients’ ages, time variables and other personally identifiable information.
